# Spread Patterns of Antibiotic Resistance in Faecal Indicator Bacteria Contaminating an Urbanized Section of the Brda River

**DOI:** 10.1007/s00248-020-01624-4

**Published:** 2020-10-24

**Authors:** Łukasz Kubera

**Affiliations:** Faculty of Biological Sciences, Department of Microbiology and Immunobiology, Kazimierz Wielki University, Al. Powstańców Wielkopolskich 10, 85-090 Bydgoszcz, Poland

**Keywords:** Antimicrobial resistance, Faecal bacteria, *Escherichia coli*, Enterococci, River pollution

## Abstract

This paper presents the spatio-temporal distribution of faecal indicator bacteria (FIB) in the river section subject to anthropogenic stress and describes spread patterns of antibiotic resistance in the studied bacterial groups. The analysis involved 58 strains of *Escherichia coli* and 61 strains of enterococci. Antibiotic resistance profiles were prepared in accordance with the recommendations of the European Committee on Antimicrobial Susceptibility Testing (EUCAST). The results indicated a correlation between the location of a sampling site and the concentration of faecal bacteria. The highest average concentrations were recorded at the site located in the city centre, where the river is used mainly for recreation. Antibiotic resistance profiles showed that *Escherichia coli* had 100% sensitivity to tigecycline, levofloxacin and imipenem. The highest percentaage of strains (17%) were resistant to piperacillin. Enterococci were 100% sensitive to levofloxacin. No strains were vancomycin-resistant (VRE). The highest percentage of strains was resistant to imipenem (23%), and the lowest, to ampicillin (2%). The spatio-temporal distribution of antibiotic-resistant strains (ARS) indicated a high concentration of drug-resistant *Escherichia coli* (47%) in the summer season at the sampling site located in the last part of the river. At the same time, drug resistance in enterococci increased along the river course and was considerably higher in spring. There were no significant relationships between physico-chemical parameters of water and the levels of faecal bacteria. On the other hand, strong relationships were observed between the percentage of strains showing resistance to the applied antibiotics and physico-chemical and biological parameters of water. The percentage of antibiotic resistant strains of *Escherichia coli* was negatively correlated with dissolved oxygen concentration (*r* = − 0.9; *p* < 0.001) and BOD_5_ (*r* = − 0.85; *p* < 0.05). The percentage of antibiotic resistant strains of enterococci was most strongly correlated with water pH (*r* = − 0.92; *p* < 0.001).

## Introduction

Since the beginning of human settlement, rivers have played a key role in the development of towns and cities, ensuring the progress of civilization. Extremely important for agriculture and transport, they were also the recipient of municipal sewage [1]. Nowadays, aquatic ecosystems in urban areas are used mainly for recreation, contirbuting to the development of tourism [2]. As a result, they are often exposed to physical, chemical and biological contamination [3]. In summer, urban aquatic ecosystems are frequently used for bathing. However, places that are not officially established as bathing areas are not regularly monitored. The recreational use of such waterbodies against the official recommendations may lead to their ecological degradation and is an epidemiological threat. Another reason for microbiological monitoring of city watercourses is that they are often used by animals, and this can affect water quality, promoting the transmission of pathogenic microorganisms [4, 5]. Since determining the concentration of all pathogens in water is simply impossible, the concentration of faecal indicator bacteria (FIB) is measured to detect and estimate the level of water contamination primarily with *Escherichia coli* and enterococci [6–8]. Nowadays, there is a growing concern about antimicrobial resistance of bacteria. The widespread use of antimicrobials in both medicine and agriculture has caused a dramatic increase in the number of drug-resistant bacterial strains in the natural environment. In surface waters, antibiotics are found at concentrations that significantly affect the activity of bacterial genes, leading, among others, to their increased mutation frequency [9]. This carries the risk of transferring resistance genes between pathogenic and non-pathogenic bacterial cells, and thus poses a direct danger to people [10]. Bacteria displaying resistance to nearly all available antibiotics were already found in anthropogenically impacted environments more than a decade ago [11].

Therefore, the present study is aimed at determining the concentration of faecal bacteria in the last, anthropogenically affected section of the Brda River and at evaluating their antimicrobial resistance. In addition, a number of environmental factors that may contribute to this increased drug resistance were identified and analysed.

## Materials and Methods

### Study Area

Located in the northern part of Poland, the Brda River is the left tributary of the Vistula, the longest river in Poland. It has the length of nearly 240 km and catchment area of approximately 4655.0 km^2^. The river flows through a patchwork of open fields and forest land in a predominantly agricultural setting. The last section cuts through the city of Bydgoszcz, where it is used for recreational purposes [12]. Bydgoszcz population is about 355,000, with 2000 people per square kilometre on average.

### Sampling Strategy

**Fig. 1:**
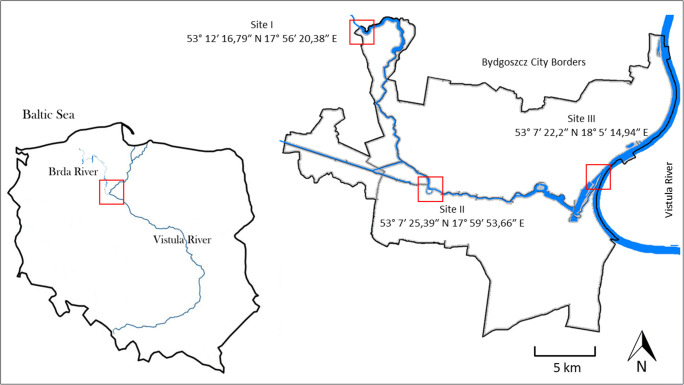
Arrangement of the sampling sites. The rivers are marked in blue. The sampling sites are marked in red

Three sampling sites were located in the studied area: (I) on the north-western border of the city, just before the river enters the city; (II) in the city centre, where the river is extensively used for recreation; (III) in the north-eastern part of the city, just before the confluence of the Brda and the Vistula. Between sampling sites I and II, there is a water pumping station. The distance between the sites was about 12 km. The location of sampling sites is presented in Fig. 1. Sampling was conducted in a seasonal cycle: in spring (21.05.2018), summer (06.08.2018) and autumn (15.10.2018). Water from the mid-point of the river, depth of about 0.5 m, was collected in triplicate to sterile bottles. The samples were immediately transported to the laboratory and analysed.

### Physico-Chemical Parameters of Water

Physico-chemical parameters of water, including pH, temperature (T), electrolytic conductivity (EC), and oxygen concentration (OC), were measured in situ using field probes (Mettler Toledo™). The results are presented in Table 1.

**Table 1:** Physico-chemical parameters of water. Results are presented as means ± standard deviation

Site	Season	T (°C)	pH	EC (μS cm^−1^)	OC (mg dm^−3^)
I	Spring	13.97 ± 0.15	7.21 ± 0.56	362.67 ± 2.52	5.39 ± 0.04
	Summer	18.67 ± 0.06	7.41 ± 0.57	334 ± 1	5.04 ± 0.08
	Autumn	15 ± 0.14	7.25 ± 0.21	311 ± 5.66	5.16 ± 0.43
II	Spring	16.73 ± 0.23	7.92 ± 0.09	380 ± 2	5.78 ± 0.05
	Summer	19.43 ± 0.15	7.91 ± 0.06	372.33 ± 3.21	5.86 ± 0.05
	Autumn	15.1 ± 0	7.9 ± 0.05	341.5 ± 4.95	4.77 ± 0.97
III	Spring	17.53 ± 0.06	7.96 ± 0.03	400 ± 1	5.69 ± 0.04
	Summer	19.5 ± 0.1	7.95 ± 0.06	387 ± 3.61	6.08 ± 0.64
	Autumn	16.95 ± 0.07	7.93 ± 0.06	232.5 ± 6.36	5.59 ± 0.17

*T* temperature, *EC* electrolytic conductivity, *OC* oxygen concentration

### Evaluation of Biochemical Oxygen Demand

The biochemical oxygen demand (BOD_5_) was measured to assess water quality at using the respirometric method and the OxiTop® Control system. This method is based on the measurement of the oxygen consumption by microorganisms during the decomposition of organic substances in a sealed sample, which is accompanied by a pressure change. Differences in oxygen consumption and carbon dioxide production resulting from the respiratory activity of bacteria are measured by OxiTop-C heads. The OxiTop OC 110 remote controller collects and interprets data using the built-in algorithm. To determine the in situ respiratory activity of planktonic bacteria, 250 mL of river water from each sampling site was poured into dark OxiTop bottles with a capacity of 500 mL. Then, 5 drops of NTH 600 nitrification inhibitor and a magnetic stirrer were put in the bottles. After a rubber quiver with two tablets (about 0.4 g) of sodium hydroxide (carbon dioxide absorber) was placed in each bottle neck, OxiTop-C measuring heads were tightly screwed on. The parameters were set on the controller, and the bottles were placed on a magnetic stirrer and incubated for 5 days at + 20 °C. The results are expressed as mg O_2_ L^−1^.

### Total Concentration of Faecal Bacteria

*Escherichia coli* were calculated according to ISO 9308-1:2014, while enterococci, to ISO 7899-2:2000. ChromoCult® Coliform Agar (CCA) selective medium was used to isolate *Escherichia coli*, while Slanetz and Bartley (SB) medium to detect and enumerate enterococci. In both cases, the membrane filtration method was used. The selected volumes of water samples were filtered through white filters with 47 mm diameter and 0.45 μm pore size. The plates with filters were incubated for 24–48 h at 35 ± 1 °C. After that, grown colonies were counted and the concentration of faecal bacteria was expressed as CFU 100 mL^−1^.

### Antimicrobial Resistance Assessment

Antibiotic resistance of faecal bacteria was determined using the disk diffusion method. *Escherichia coli* isolated from the CCA medium and enterococci isolated from the SB medium were used for the analysis. Strains used for the preparation of drug sensitivity profiles were checked for purity using API® 20E and API® 20 Strep test strips (bioMérieux, France). Antimicrobial resistance of *Escherichia coli* isolated (*n* = 58) from CCA medium and enterococci isolated (*n* = 61) from SB medium was determined using the disk diffusion method. Mueller-Hinton agar was inoculated with the volumes of 0.1 mL of bacterial suspension equivalent to 0.5 McFarland turbidity standard. Subsequently, paper disks soaked with selected antibiotics were placed on the Petri dishes. After 18 h of incubation at 36 °C, zones of inhibited growth were measured and the results were compared with the guidelines of the European Committee on Antimicrobial Susceptibility Testing [13]. Characteristics of used antibiotics are presented in Table 2.

**Table 2:** Characteristic of used antibiotics

Class of antibiotic	Type of antibiotic	Symbol of disk	Concentration (μg)
Carbapenems	Imipenem	IMP	10
Fluoroquinolones	Levofloxacin	LEV	5
Tetracyclines	Tigecycline	TGC	15
Glycopeptides	Vancomycin^a^	VA	5
Penicillins	Ampicillin^a^	AM	2
	Piperacillin^b^	PRL	30
Cephalosporins	Cefoxitin^b^	FOX	30
Aminoglycosides	Gentamicin^b^	CN	10

^a^Only for enterococci

^b^Only for *Escherichia coli*

### Statistical Analysis

Due to the fact that the data were not normally distributed, the analyses were conducted using nonparametric tests. The analysis of difference in faecal indicator bacteria count between research sites and seasons was based on the Kruskal-Walis test (one-way ANOVA on ranks). Relationships between physico-chemical parameter values and the counts of faecal indicator bacteria as well as ARS strains were determined using Spearman correlation coefficient. All statistical tests had a significance level *p* ≤ 0.05.

## Results

**Fig. 2:**
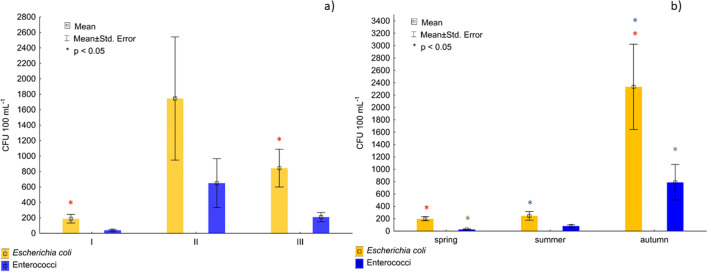
Average number of faecal indicator bacteria **a** at the research sites, **b** in the research seasons. Statistical differences within groups of data was based on post hoc for Kruskal-Wallis test (*p < 0.05)

Faecal bacteria are the basic indicator of sanitary water quality in aquatic ecosystems. According to the results, all water samples contained faecal bacteria. Their highest average concentration was recorded at sampling site II located in the city centre (1744 CFU 100 mL^−1^ for *Escherichia coli* and 650 CFU 100 mL^−1^ for enterococci), while the lowest, at sampling site I, i.e. before the river enters the city (189 CFU 100 mL^−1^ for *Escherichia coli* and 39 CFU 100 mL^−1^ for enterococci) (Fig. 2a). The concentration of faecal bacteria depended on the season of the year; while it was similar in spring and summer, it increased considerably in autumn, reaching 2333 CFU 100 mL^−1^ in case of *Escherichia coli* and 789 CFU 100 mL^−1^ in case of enterococci (Fig. 2b). Statistical analysis indicated a strong relationship between both the location and season of the year and the concentration of the studied bacterial groups. Significant differences in the concentration of *Escherichia coli* (*p* < 0.05) were noted between sampling sites I and III and between spring/summer and autumn. The season of the year also affected the distribution of enterococci, with significant differences between spring and autumn.

Biological oxygen demand ranged from 1.8 mg O_2_ L^−1^ at sampling site I in autumn to 43.4 mg O_2_ L^−1^ at sampling site III in spring. Although BOD_5_ values increased along the river current, the differences were not statistically significant. On the other hand, a strong relationship was observed between the season of the year and the distribution of BOD_5_: the highest average values were recorded in spring, while the lowest, in autumn. Multiple comparison tests indicated differences between these two seasons at the significance level *p* < 0.0001 (Table 3).

**Table 3:** Results of BOD_5_ values expressed as mg O_2_ L^−1^ and presented as means ± standard deviation. Statistical differences within groups of data was based on post hoc for Kruskal-Wallis test

Season	Sampling sites		
	I	II	III
Spring**	27.19 ± 14.09	29.83 ± 8.15	43.40 ± 0.00
Summer*	18.97 ± 12.43	21.70 ± 5.40	26.23 ± 7.85
Autumn*^,^**	1.80 ± 1.56	3.60 ± 1.56	4.53 ± 5.68

*p < 0.05; **p < 0.0001

**Fig. 3:**
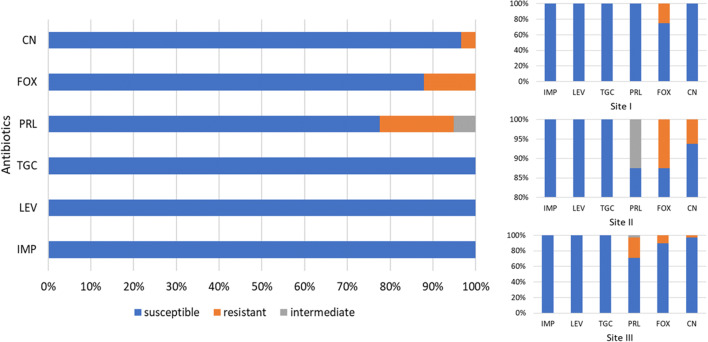
Antimicrobial resistance of *Escherichia coli* strains (*n* = 58) divided into sampling sites. CN gentamicin, FOX cefoxitin, PRL piperacillin, TGC tigecycline, LEV levofloxacin, IMP imipenem

**Fig. 4:**
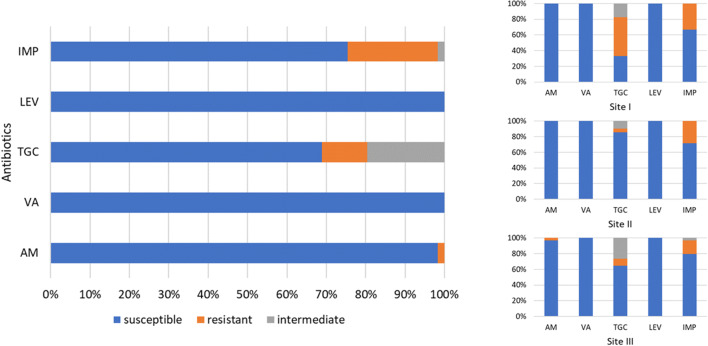
Antimicrobial resistance of enterococci strains (*n* = 61) divided into sampling sites. IMP imipenem, LEV levofloxacin, TGC tigecycline, VA vancomycin, AM ampicillin

Drug resistance profiles indicated that the majority of faecal strains were sensitive to the applied antibiotics. *Escherichia coli* (Fig. 3) were 100% sensitive to tigecycline, levofloxacin and imipenem. The resistance to other antibiotics varied. The highest percentage of strains (17%) showed resistance to piperacillin, the only antibiotic to which 5% of the strains were medium sensitive. Then, 12% of the strains were resistant to cefoxitin, and 3% to gentamycin. All enterococci isolates (Fig. 4) showed complete sensitivity to levofloxacin and vancomycin. The highest number of enterococci were resistant to imipenem (23%), while the lowest to ampicillin (2%). Eleven percent of enterococci isolates showed resistance to tigecycline, while 20% were medium sensitive.

**Fig. 5:**
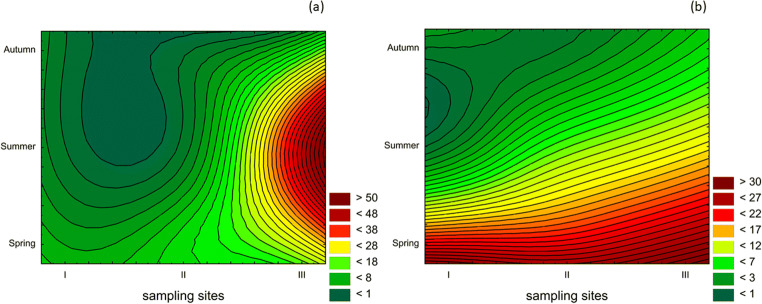
Spatio-temporal percentage distribution of antibiotic resistant strains (ARS): **a** *Escherichia coli* (*n* = 17); **b** enterococci (*n* = 22)

Figure 5 presents spatio-temporal distribution of strains which were resistant to at least one of the applied antibiotics. A high accumulation of antimicrobial-resistant strains of *Escherichia coli* strains (47%) was recorded in summer at sampling site III, the last part of the Brda River (Fig. 5a). At the same time, the number of resistant strains of enterococci increased along the river current and was highest in spring (Fig. 5b). None of the significant differences between the number of antibiotic resistant strains and sampling sites as well as between seasons were noted.

The study also examined the impact of selected environmental parameters on the concentration of faecal bacteria and the percentage of antibiotic resistant strains (ARS). As can be seen from Table 4, none of the parameters was significantly correlated with the total bacterial concentration. However, strong correlations were noted between the physico-chemical and biological parameters of water and the percentage of strains showing resistance to the tested antibiotics, e.g. the percentage of antimicrobial-resistant strains of *Escherichia coli* was negatively correlated with dissolved oxygen concentration (*r* = − 0.9; *p* < 0.001) and BOD_5_ (*r* = − 0.85; *p* < 0.05). Similarly, there was a strong relationship between low oxygen concentration and the percentage of antibiotic-resistant strains of enterococci (*r* = − 0.73; *p* < 0.05). However, the percentage of antibiotic-resistant enterococci isolates was most strongly correlated with water pH (*r* = − 0.92; *p* < 0.001).

**Table 4:** Correlations between physico-chemical and biological parameters of water and total number of faecal bacteria including antibiotic resistance strains based on Spearman correlation coefficient

	T (°C)	pH	EC (μS cm^−1^)	OC (mg dm^−3^)	BOD_5_
	Correlation (*r*)				
*Escherichia coli*	− 0.13	0.37	− 0.16	− 0.08	− 0.40
ARS	− 0.55	− 0.58	− 0.82*	− 0.90**	− 0.85*
Enterococci	0.15	0.27	− 0.20	− 0.32	− 0.27
ARS	− 0.53	− 0.92**	− 0.70*	− 0.73*	− 0.66

*T* temperature, *EC* electrolytic conductivity, *OC* oxygen concentration, *BOD*_*5*_ biochemical oxygen demand, *ARS* antibiotic resistance strains

*p < 0.05; **p < 0.001

## Discussion

Rivers are dynamic, constantly evolving ecosystems, which undergo numerous changes as a result of natural processes and human activity. Since anthropopressure has such a significant impact on water quality in aquatic environments [14], a microbial assessment is necessary to reduce the risk from waterborne pathogens [6]. Moreover, high levels of faecal bacteria in surface waters, a common problem in urban areas, reduce their recreational values [15]. Microbiological analysis of the Brda River, flowing through a city of 350,000, showed that the concentration of faecal bacteria depended on both the location of sampling sites and seasons of the year. The highest average concentrations of *Escherichia coli* and enterococci were recorded in cold and rainy autumn months. This dynamic increase could have resulted from different meteorological conditions after warm and sunny summer. Cho et al. [16] presented a model showing that sunlight and precipitation are key factors affecting FIB, with drought and strong sunlight limiting, and intensive rainfall, accelerating their growth. These relationships have also been confirmed by other researchers [17, 18]. The analysis of the spatial distribution of faecal bacteria indicated that both *Escherichia coli* and enterococci were the least abundant at the sampling site located at the beginning of the studied section of the river. The average maximum values were recorded in the city centre and slightly lower, in the last part of the river. Although the concentration of FIB depended significantly on the location of sampling sites (*p* > 0.05), it did not increase along the river course. The observed growth at sampling site II was probably related to increased recreational activity in the city centre, featuring a beach and a water sports centre. However, high FIB concentration in this particular place can be attributed not only to the presence of people, but also animals. This part of the Brda River plays an important ecological role, providing habitat for many species of animals, including birds. Strauch [19], examining the impact of ecological factors on microbial quality of rivers in the Serengeti National Park in Tanzania, also maintained that animals could be the primary source of faecal bacteria in surface waters, which, especially in view of the increased demand for water resources, can pose a serious threat to human health. Nguyen et al. [20] found a positive correlation between FIB and a bird marker gene and pointed out that birds, not sewage, were the main source of faecal bacteria in examined samples. Although seasons of the year and changing weather conditions are two key factors affecting the concentration of faecal bacteria in surface waters, there are other environmental variables that can play an important role as well. Many studies have emphasized a correlation between FIB concentration and physico-chemical parameters of water. In the Betna River [21], the concentration of faecal bacteria was positively correlated with rainfall and water temperature. On the other hand, the results obtained by Wang et al. [22] showed no significant relationship between the levels of *Escherichia coli* and enterococci and any of the eleven physico-chemical parameters of water in the rivers of the Haihe river basin (China). In the present study, no significant influence of measured parameters on the size of FIB population was observed. Biochemical oxygen demand is among the most common criteria for assessing water quality [23]. It provides information to what extent a waterbody is contaminated with biodegradable organic matter [24]. High BOD loadings in freshwater ecosystems come mainly from anthropogenic sources, such as household and animal waste, industrial waste, and municipal sewage [25]. This may explain the relationship between *Escherichia coli* and BOD_5_. Thus, it can be concluded that for the studied section of the Brda River, the season and location of the sampling site were the only variables that significantly affected the growth of FIB. As has been already emphasized, aquatic ecosystems should be systematically monitored for sanitary water quality due to the potential epidemiological risk associated with the presence of faecal bacteria. This is of particular importance considering the functions of these ecosystems in urban environments and interactions between people and these ecosystems [26]. However, in the modern era of antibiotc abuse microbiological monitoring may be an insufficient measure. One of the key strategies to combat this increased antimicrobial resistance is to monitor the use of antibiotics and the spread of antimicrobial resistance [27]. Not only do antibiotic-resistant bacteria in natural waters pose a serious threat to humans and animals but they also impair the ecosystem functioning. Drug-resistant bacteria can affect the structural and functional diversity of microbial communities that play a key role in many ecological processes, particularly in those related to the maintenance of water quality [28]. My results suggested varied drug resistance among the studied FIB strains. *Escherichia coli* had the highest resistance to piperacillin (17%), which is probably associated with their production of beta-lactamases that break the antibiotic structure. The production of these enzymes by *Escherichia coli* isolated from aquatic environments has been confirmed by many researchers [29–31]. All investigated *Escherichia coli* strains were sensitive to tigecycline, levofloxacin and imipenem. Resistance to the latter is very rare in environmental isolates [32]. In the study by Swedan and Alrub [33], *Escherichia coli* isolated from the sources of drinking water were also completely sensitive to imipenem and tigecycline. On the other hand, enterococci showed high resistance to imipenem: it was observed in 23% of the strains. However, no vancomycin-resistance strains (VRE) were identified. Veljović et al. [34] also observed complete sensitivity to vancomycin among enterococci isolated from aquatic ecosystems. At the same time, Alipour et al. [35] recorded vancomycin resistance only in 3 of 70 bacterial isolates representing four enterococcal species obtained from the aquatic environment. There were certain differences in spatio-temporal distribution of strains showing resistance to at least one of the applied antibiotics between the investigated bacterial groups. Antibiotic-resistant strains of *Escherichia coli* dominated in summer, with the highest percentage recorded in the lower course of the studied river section. On the other hand, antibiotic-resistant strains of enterococci dominated in spring, and their percentage increased gradually along the river course. Suzuki et al. [36], while examining resistance acquisition of *Escherichia coli* in the river environment, also observed an increased percentage of resistant strains at sampling sites located in the lower river course. Cho et al. [37] emphasize that antibiotic resistance is one of the biggest public health challenges of our time; therefore, more attention should be paid to the emergence and spread of antimicrobial resistance in aquatic environments. There is also a need for a better understanding of this phenomenon in humans and animals. Jia et al. [38] suggest that river ecosystems are natural reservoirs of antibiotic resistance genes, and that human activity contributes to the persistence of antibiotic contamination. Although research indicates that the natural environment is an important element in the transmission of resistant bacteria, these processes still need more recognition, especially at the ecological level [39]. Correlation analysis showed that although the measured environmental parameters did not affect the size of the faecal indicator bacteria population, they might have played a significant role in the development of their antibiotic resistance. Statistically significant negative correlations between the emergence of resistant strains and environmental variables such as oxygen concentration and electrolytic conductivity were recorded for both *Escherichia coli* and enterococci. Moreover, a strong relationship between water pH and the occurrence of resistant enterococcal strains was noted. So far, few studies have investigated the impact of environmental parameters on the increase of bacterial resistance in aquatic environments. Harnisz [40], who examined changes in resistance among indigenous bacteria in the river receiving treated sewage, also noticed negative correlations between water pH and oxygen concentration and antibiotic-resistant bacteria. Fletcher [41] maintains that understanding environmental factors is necessary to limit or prevent the spread of antibiotic resistance in the environment. Therefore, future research should confirm the observed relationships and determine whether environmental parameters can contribute to the development of mechanisms of antimicrobial resistance transmitted both vertically and horizontally.

## Conclusion

In the current study, the faecal concentrations were correlated mainly with the season of the year and the location of sampling sites. Maximum concentrations were recorded in rainy autumn months at the sampling site affected by anthropopressure to the highest degree, which corresponds with the results of other studies. According to the prepared drug resistance profiles, the majority of faecal bacteria were sensitive to the applied antibiotics. The strains with varied resistance to the selected antimicrobials had different spatial distribution. Moreover, the results suggested the potential relationship between physico-chemical and biological parameters of water and increased drug resistance among faecal indicator bacteria. Further research, with the use of molecular tools, should be carried out to examine whether certain environmental variables can induce the expression of genes responsible for triggering antibiotic resistance in bacteria.
